# 

**Published:** 1915-11

**Authors:** 


### CONTENTS.

ORIGINAL COMMUNICATIONS—

Variety of Work in a Base Military Hospital, by Marion C. Pruitt, L. R. C. P., S., M. D., Atlanta, Ga. ........ 365

Some Facts and Fiction That Will Stimulate Atlanta Doctors to Activity in Medico-Social Refirm, by C. C. Aven, M. D. ........ 368

Report of Five Hundred and Seventy Cases Treated in the Military Hospital American Red Cross, Kiev, Russia, by Dean F. Winn, M. D., Atlanta, Ga. ........ 373

Notes on Psychoanalysis, by Hansell Crenshaw, M. D., Atlanta, Ga. ........ 388

Child Birth—Early Rising After, by E. C. Cartledge, M. D. ........ 391

"The Abuse of Pituitrin, Intra-Partum," by F. J. Giuffrida, M. D., Atlanta, Ga. ........ 394

EDITORIAL ........ 396

SELECTIONS AND ABSTRACTS ........ 402

BOOK REVIEW ........ 408

